# 

**Published:** 1896-09-12

**Authors:** 


					THE HOSPITAL, ABSOLUTELY PURE, therefore BEST,
Sept. 12th, 1896.
CADBURY'S u ^ CADBURY'S
COCOA imI\ M cocoa
m> Mm> >??L> NO ALKALIES USED.
tAscoff Westerh Infirmary ' - ^'"'^wKFSuC^omlamwh hospltaj.
No. 520. Y?i. xx. WITH EXTRA NURSING SUPPLEMENT. WSE'
Satttrr> av St?pt 1 9ttt 1 RQfi [Registered at the General Post Office as a Newspaper for Monthly Parts, 6d.; post free,8<l
I ' i 1 transmission in the United Kingdom and Abroad.] Ditto per Annum, 8s. 6d., post free

				

